# Capacity-Delay Trade-Off in Collaborative Hybrid Ad-Hoc Networks with Coverage Sensing

**DOI:** 10.3390/s17020232

**Published:** 2017-01-26

**Authors:** Lingyu Chen, Wenbin Luo, Chen Liu, Xuemin Hong, Jianghong Shi

**Affiliations:** 1 Department of Communications Engineering, School of Information Science and Technology, Xiamen University, Xiamen 361005, Fujian, China; chenly@xmu.edu.cn (L.C.); 23320151154076@stu.xmu.edu.cn (W.L.); 23320120153931@stu.xmu.edu.cn (C.L.); shijh@xmu.edu.cn (J.S.); 2 Key Laboratory of Underwater Acoustic Communication and Marine Information Technology, Ministry of Education, Xiamen University, Xiamen 361005, Fujian, China

**Keywords:** capacity-delay trade-off, ad hoc network, device-to-device

## Abstract

The integration of ad hoc device-to-device (D2D) communications and open-access small cells can result in a networking paradigm called hybrid the ad hoc network, which is particularly promising in delivering delay-tolerant data. The capacity-delay performance of hybrid ad hoc networks has been studied extensively under a popular framework called scaling law analysis. These studies, however, do not take into account aspects of interference accumulation and queueing delay and, therefore, may lead to over-optimistic results. Moreover, focusing on the average measures, existing works fail to give finer-grained insights into the distribution of delays. This paper proposes an alternative analytical framework based on queueing theoretic models and physical interference models. We apply this framework to study the capacity-delay performance of a collaborative cellular D2D network with coverage sensing and two-hop relay. The new framework allows us to fully characterize the delay distribution in the transform domain and pinpoint the impacts of coverage sensing, user and base station densities, transmit power, user mobility and packet size on the capacity-delay trade-off. We show that under the condition of queueing equilibrium, the maximum throughput capacity per device saturates to an upper bound of 0.7239 $\lambda_{b}/\lambda_{u}$ bits/s/Hz, where $\lambda_{b}$ and $\lambda_{u}$ are the densities of base stations and mobile users, respectively.

## 1. Introduction

Exploitation of the spatial domain is a primary way to address the challenge of exponential capacity demand in cellular communication networks [1]. Small cells [2] and device-to-device (D2D) communications [3,4] are both effective solutions to enhance the cellular network capacity by increasing the spatial reuse factor of the limited spectrum. An alternative approach to address the exploding traffic challenge is to exploit the traffic delay domain. This is motivated by the fact that a large portion of mobile data traffic is consumed by content delivery, which is non-real-time in nature. Unlike real-time services that have strict delay constraints, content delivery services have a greater flexibility to be manipulated in the delay domain (e.g., by proactive content pushing) [5,6]. It has been shown that relaxed delay constraints can be traded for capacity. This drives an emerging research field of content-centric mobile communications, which aim to find capacity-efficient solutions for massive content delivery [7,8,9].

The integration of ad hoc D2D communications and open-access small cells can result in a fundamental networking paradigm called the hybrid ad hoc network, which is a promising paradigm for future mobile communication networks. The objective of this paper is to investigate the fundamental trade-off between capacity and delay in such hybrid ad hoc networks. The capacity study of cellular D2D networks can take the reference from the extensive literature on the capacity of wireless ad hoc networks. Most existing works in this field have adopted a popular information-theoretic framework called scaling law analysis. Gupta and Kumar first proposed this framework and showed that the per-node transport capacity of arbitrary static ad hoc networks scales as $1/\sqrt{n}$ [10], where *n* is the number of nodes in the network. This result suggests that the capacity of each node diminishes as *n* goes large. Subsequent works on static ad hoc networks, such as [11,12,13,14], all lead to similar pessimistic results.

Based on an important insight that mobility can be exploited to enhance capacity at the expense of increased delay, Gorssglauser and Tse [15] showed that in mobile ad hoc networks, a constant per node throughput can be achieved with a two-hop relaying scheme. Several subsequent works have studied the amount of delays required to achieve a level of capacity for various mobility models, such as i.i.d. mobility [16], random walk [17,18,19], Brownian motion [20] and Levy walk [21,22]. The delay required for constant per node throughput has been shown to scale as fast as the network size.

Apart from mobility, it has been shown that adding infrastructure (e.g., base stations (BSs)) to pure ad hoc networks, resulting in the so-called hybrid wireless networks, can bring significant benefits in terms of capacity and delay. The capacity of hybrid networks with static nodes has been studied in [23,24,25,26,27,28]. It was shown that capacity increases linearly with the number of BSs, given that the number of BSs grows faster than $\sqrt{n}$ [28]. In [29], it is shown that a constant delay can be achieved. The capacity scaling law of hybrid networks with mobile nodes is studied in [30], where some mobility-dependent extra gains on the capacity are shown. The study of capacity-delay trade-off using the “scaling law” analysis has attracted much research attention in recent years. Research has been extended to address various aspects, such as motion-cast [31,32], multi-cast [33,34,35,36], converge-cast [37], group and correlated mobility [38,39,40,41,42,43], cognitive radio [44,45], etc.

Despite the enormous success and popularity of the “scaling law” framework, this framework also has some limitations. First, for a tractable analysis, the “protocol model” [10] is usually assumed to describe the communication and interfering range of a transmitter. This model, however, does not take into account accumulated interference, which can become significant in dense networks. Second, the delays incurred by buffering and queueing are commonly neglected for simplicity, resulting in potentially under-estimated delays. For example, consider a mobile node with a large amount of buffered data and a short time opportunity to access a BS. It is likely that some buffered data cannot be delivered in the first access opportunity and should wait for the next chance. As a result, queueing delays are coupled with mobility-related delays, which can potentially lead to a significant increase of the overall delay. It should be noted that the delay we considered in this paper is the fundamental delay caused by ideal (i.e., infinite-buffer) queueing at the physical layer. This delay is different from other studies that considered specific medium access control (MAC) layer functions, such as retransmission schemes [46,47]. Third, previous studies have mostly focused on the average measures, e.g., the mean delays. Such an average measure can be misleading in the case of long-tail distributions, in which the mean is biased by infrequent incidents of very large values. Because long-tail delay distributions are common in communication networks, it is very desirable to gain finer-grained insights into the exact distribution of delays.

To address the above limitations, this paper proposes an alternative analytical framework based on queueing theoretic models and physical interference models. Although both models have been used extensively for the performance study of wireless networks, the effort to unify both models in a coherent framework is still rare. Our previous conference paper [48] was an early attempt to propose a unified framework for the performance analysis of hybrid ad hoc networks. The basic idea is to capture the stochastic phenomenon of user mobility and coverage outage using queueing dynamics. However, the work was still incomplete and does not consider the issue of multi-user access. This paper further extends and refines the unified framework and provides comprehensive analysis. Specifically, new issues, including multi-user access, capacity limit and power and rate optimization, are addressed in this paper. The new framework allows us to fully characterize the delay distribution in the transform domain and pinpoint the impacts of user and BS densities, transmit power, user mobility and packet size on the uplink capacity-delay trade-off. We reach a conclusion that the maximum throughput capacity per user is bounded by 0.7239 $\lambda_{b}/\lambda_{u}$ bits/s/Hz, where $\lambda_{b}$ and $\lambda_{u}$ are the densities of base stations and mobile users, respectively.

The remainder of this paper is organized as follows. The system model is described in Section 2. Section 3 introduces the new analytical framework combining both queueing models and physical interference models. A detailed characterization of delay distributions and the fundamental limits of per node capacity throughput are presented in Section 4. In Section 5, we discuss the aspects of rate and power optimization to achieve the minimum average delay. Finally, numerical results are presented in Section 6, and conclusions are drawn in Section 7. For the convenience of the readers, the major parameters defined in this paper are summarized in the table (Table 1) below.

## 2. System Model

We consider a hybrid ad hoc network with small cells and mobile users. We are interested in the uplink scenario, where mobile users transmit message to the small cell BSs. The small cell BSs are randomly deployed following two-dimensional homogeneous Poisson point processes (PPPs) with density $\lambda_{b}$. We assume that a single dedicated frequency band is used by all small cells to provide best-effort coverage in the presence of self-interference. Similarly, the mobile users are assumed to be randomly deployed following a PPP with density $\lambda_{u}$. Each user has a homogeneous throughput capacity demand of *C* bits/s. More specifically, we assume that each user has an incoming traffic stream with fixed packet size *L*. It is assumed that all users have identical and random mobility patterns, so that they randomly move in and out of the small cell coverage areas from time to time. The average speed of a user is denoted by *v*. The duration when a user is not in coverage is called coverage outage.

### 2.1. User Collaboration Protocol

**Figure 1:**
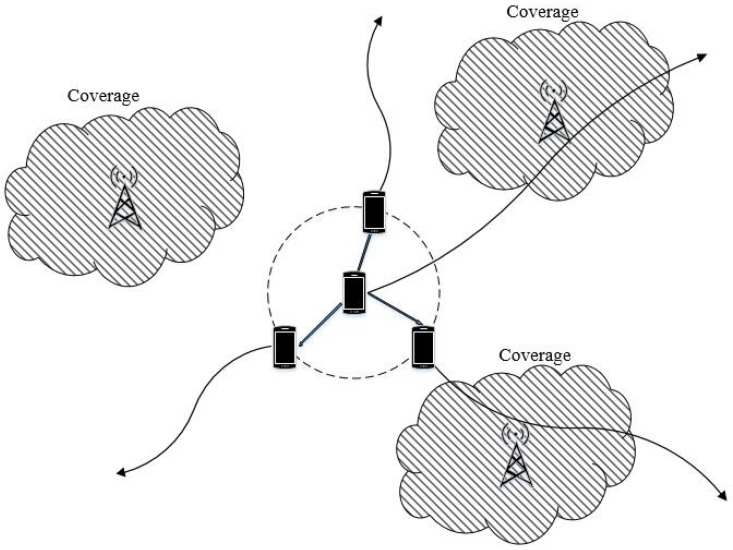
System model of the hybrid ad hoc network with user collaboration and coverage sensing.

As illustrated in Figure 1, we consider a user collaboration scheme with two-hop decode and forward relay. This simple scheme was frequently assumed in the literature and has been shown to achieve the optimal scaling in mobile ad hoc networks [15]. Our study focuses on the uplink access scenario, which includes two phases: broadcast phase and deliver phase.

- In the broadcast phase, original packets on a device are broadcast in the D2D band with a constant rate $R_{I}$ and constant power $P_{I}$. Nearby users who can successfully decode the packet will store the packet. Each packet is broadcast only once from its original user.
- In the deliver phase, the original traffic and traffic received from other users during the broadcast phase are buffered in a queue and wait to be transmitted to a BS. A transmission to the BS starts only when a packet carrier falls within the coverage of a small cell. The packets are transmitted following a first-come-first-out (FIFO) policy until the buffer empties or a coverage outage occurs. The transmit power and rate used to communicate with the BSs are denoted as $P_{\mathrm{II}}$ and $R_{\mathrm{II}}$, respectively. Once the transmission of the first copy of a packet starts, a signaling is performed so that all other copies of the same packet will be dropped [15]. In cases that a packet transmission is interrupted by a coverage outage, the transmission will be resumed to transmit the rest of the packet once the user moves into coverage again. In other words, we assume a preemptive-resume queueing policy, noting that our results can be easily extended for a similar preemptive-repeat policy.

The frequency bands used for D2D communications and small cell access are different to avoid interference. Without loss of generality, we assume that both frequency bands have the same normalized bandwidth of one.

### 2.2. Interference Model

Whether a user is within the coverage of a small cell transmitter is determined by its received signal-to-noise-and-interference ratio (SINR). Unlike “protocol models” [10] that use two idealistic circles to represent the transmit range and interfering range of a transmitter, in this paper, we consider the physical interference model, which considers the accumulation of interference from multiple transmitters. Consider a random field of non-collaborative transmitters distributed as a two-dimensional PPP process and transmitting with identical power *P*; the receive SINR at a typical (randomly chosen) location is given by:

(1) $$\gamma =\frac{Ph}{PI+1}$$

where *P* is the transmit power normalized to the Gaussian noise power, *I* is the accumulated interference normalized to *P*, *h* is the channel gain given by $h=gd^{-\eta }$, *d* is a random variable (RV) denoting the distance between the active user and the inactive user, *η* is the path loss exponent and the RV g∼exp(1) follows an exponential distribution with unit mean to represent the power gain of Rayleigh fading channels. The accumulated interference *I* is given by:

(2) $$I=\sum_{i}g_{i}d_{i}^{-\eta },\;\;\;\;\;\left(i=1,2,\ldots \ldots \infty \right)$$

where *i* is the index of interfering active users, $d_{i}$ is the distance from the inactive user to the *i*-th interferer and $g_{i}∼\exp\left(1\right)$ are RVs to account for Rayleigh fading in the interference channels. According to the spatial PPP model, the PDF of *d* is given by [49]:

(3) $$f_{d}^{\prime }\left(x\right)=e^{-\lambda \pi x^{2}}2\pi \lambda x,\;\;\;x\in \left(0,\infty \right)$$

where *λ* is the spatial density of transmitters. In the context of wireless networks, the above PDF could result in an unrealistic calculation of the path loss when the common path loss model is applied. When d∈(0,1), we have $d^{-\alpha }>1$, implying that the receive power becomes greater than the total transmit power, which is unrealistic. A practical approach to reduce this inaccuracy is to limit the range of *d* as d∈[1,∞). This leads to a slightly modified PDF given by:

(4) $$f_{d}\left(x\right)=e^{\lambda \pi }e^{-\lambda \pi x^{2}}2\pi \lambda x,\;\;\;x\in \left(1,\infty \right).$$

Numerical results show that the difference between (3) and (4) becomes significant when the transmitter density becomes higher than 0.1 users/m${}^{2}$. Consider a typical receiver on the plane; the received SINR is an RV. Following a similar procedure in [50], but applying the modified PDF of *d* given by (4), the complementary CDF (CCDF) of SINR, given the path loss exponent η=4, can be derived as:

(5) $$\begin{array}{ccc} {\tilde{F}}_{\gamma }\left(x;\lambda ,P\right) & = & \pi \lambda e^{\pi \lambda }\int_{1}^{\infty }e^{-ay-by^{2}}dy \end{array}$$

(6) $$\begin{array}{ccc} & = & \frac{\pi^{3/2}\lambda e^{\lambda \pi }}{\sqrt{b}}e^{\frac{a^{2}}{4b}}Q\left(\sqrt{2b}+\frac{a}{\sqrt{2b}}\right) \end{array}$$

where:

(7) $$a=\lambda \pi \left[1+\sqrt{x}\arctan\left(\sqrt{x}\right)\right]$$

and b=x/P. In Equation (6), Q(·) denotes the Q-function. Given that λ<0.1 users/m${}^{2}$, which suits most practical scenarios, Equation (6) can be well approximated by:

(8) $${\tilde{F}}_{\gamma }^{*}\left(x;\lambda ,P\right)\approx \frac{\pi^{\frac{3}{2}}\lambda_{a}}{\sqrt{b}}e^{\frac{a^{2}}{\sqrt{2b}}}Q\left(\frac{a}{\sqrt{2b}}\right)$$

In the case of P→∞, Equation (8) can be further simplified to [50]:

(9) $${\tilde{F}}_{\gamma }^{\lim}\left(x\right)=\lim_{P\to \infty }F_{\gamma }\left(x;\lambda ,P\right)=\frac{1}{1+\sqrt{x}\arctan\left(\sqrt{x}\right)}.$$

### 2.3. Remarks on System Parameters

Summarizing the above system description, two types of parameters can be distinguished. The first type is the system parameters, including the user packet arrival rate *χ*, packet size *L*, user density $\lambda_{u}$, base station density $\lambda_{b}$ and user speed *v*. These are given parameters that cannot be optimized by protocol design. We note that the capacity per user is given by C=χL. The second type is the protocol parameters, including power parameters $P_{I}$ and $P_{\mathrm{II}}$ and rate parameters $R_{I}$ and $R_{\mathrm{II}}$. These parameters can be optimized by protocol designs.

Based on the above system description, our research objective is to gain theoretical insights into the following questions: (1) How is the distribution of packet delay related to the system and protocol parameters? (2) Given the system parameters, how can protocol parameters be optimized for delay performance? (3) Is there a fundamental limit of per node throughput capacity *C*? (4) Given optimized protocol parameters, what is the trade-off between capacity and delay? How does this trade-off change with different system parameters? Before addressing these questions, a new analytical framework is introduced to transform the above system model into a mathematically-tractable queueing model.

The following notations regarding an RV will be applied throughout the text. Given an RV denoted as *α*, we will use $\bar{\alpha }$ to denote its mean, $\hat{\alpha }$ to denote its second moment, $f_{\alpha }\left(t\right)$ to denote its probability density function (PDF), $F_{\alpha }\left(t\right)$ to denote its cumulative distribution function (CDF), ${\tilde{F}}_{\alpha }\left(t\right)$ to denote its complementary CDF and $L_{\alpha }\left(s\right)$ to denote its Laplace transform. The Laplace transform of an RV is given by:

(10) $$L_{\alpha }\left(s\right)=E\left(e^{s\alpha }\right)=\int_{0-}^{\infty }e^{st}dF_{\alpha }\left(t\right)$$

where E(·) denotes expectation.

## 3. A Queueing Model-Based Analytical Framework

Our analytical framework is based on a queueing model that characterizes the behavior of data buffering, collaborative packet delivery and random processes of coverage outage. This section will explain how parameters of the queueing model can be derived from the various system parameters and protocol parameters introduced in the previous section.

### 3.1. A Queueing Model

**Figure 2:**
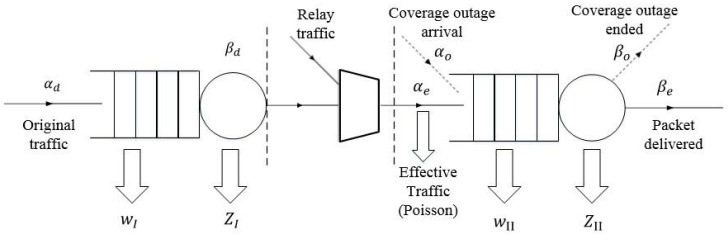
Queueing model representation of the hybrid ad hoc network.

Consider the packet transmission process in a typical device, the delays incurred in different phases can be described by the queueing model illustrated in Figure 2.

#### 3.1.1. Queueing in the Broadcast Phase

In the broadcast phase, original traffic is buffered in a device before it can be broadcast. The queue is characterized by two RVs $\alpha_{d}$ and $\beta_{d}$, which represent the random packet arrival interval and transmission time of packets, respectively. Under the assumption of fixed packet size and constant broadcast rate, $\beta_{d}$ becomes a constant given by $\beta_{d}=L/R_{I}$. Define the load parameter $\varepsilon_{d}=\bar{\beta_{d}}/\bar{\alpha_{d}}$; this parameter represents the fraction of time during which a device is active in broadcasting.

The delays incurred in this queueing process include waiting time $w_{I}$ and completion time $z_{I}$. The former is defined as the duration from the arrival of a packet till the start of its transmission. The latter is defined as the duration from the start of a packet’s transmission to the end of the transmission. Define sojourn time as $s_{I}=w_{I}+z_{I}$. This indicates the total time a packet spent in a queue.

The number of users that can successfully receive the packet from a broadcasting user is a discrete RV denoted by *N*. The probability mess function (PMF) of *N* is denoted by $f_{N}\left(n\right)$. After the broadcast, we call a packet belonging to type-*n* traffic if there are *n* copies of the packet in the system, i.e., the packet has been successfully broadcast to n−1 more users.

#### 3.1.2. Effective Traffic

Packets coming from the original traffic and packets received from other users via broadcast are buffered in a queue before they can be delivered to the BS. These packets, however, may be dropped if one of their copies gets transmitted first from other packet carriers. A rigid representation of the actual queueing process requires a complicated model involving queueing network, which is analytically intractable.

To simplify the analysis, we define “effective traffic” of a device as packets that eventually get transmitted from the device. Because users are homogeneous, the effective traffic load of a user should be the same as the original traffic load. After the broadcast phase, the average type-*n* traffic received by a user is given by $nf_{N}\left(n\right)/\alpha_{d}$. Because there are *n* copies undergoing the independent queueing process on different users, the probability that a type-*n* packet gets transmitted from a particular user is 1/n. Therefore, the effective type-*n* traffic delivered from a user becomes $f_{N}\left(n\right)/\alpha_{d}$. Summing up all of the traffic types for *n* ranging from one to ∞, it can be easily shown that the overall effective traffic load of a device equals $1/\alpha_{d}$, i.e., $\sum_{n=1}^{\infty }f_{N}\left(n\right)/\alpha_{d}=1/\alpha_{d}$. Because non-effective traffic packets are dropped before transmission as if they have never arrived on a device, only effective traffic will contribute to the actual queueing delays.

#### 3.1.3. Queueing in the Deliver Phase

A preemptive-resume priority queueing model is used to describe the queueing behavior in the deliver phase. This model assumes two classes of independent traffic. The first class represents the coverage outage process, while the second class represents the effective traffic. The first class has absolute priority over the second class. This means that once a coverage outage occurs, the current transmission is stopped and should wait till the next coverage opportunity.

The effective traffic is characterized by two random RVs $\alpha_{e}$ and $\beta_{e}$. The former characterizes the arrival intervals of effective traffic packets, while the latter characterizes the uninterrupted transmission time of a packet. The outage process is characterized by $\alpha_{o}$ and $\beta_{o}$. The former represents the random duration between the arrivals of two outages, while the latter describes the random duration of an outage. Define load parameters $\varepsilon_{e}={\bar{\beta }}_{e}/{\bar{\alpha }}_{e}$ and $\varepsilon_{o}=\bar{\beta_{o}}/\bar{\alpha_{o}}$. The combined load of the two classes of traffic is $\varepsilon_{\mathrm{II}}=\varepsilon_{e}+\varepsilon_{o}$, and a stable queue requires $\varepsilon_{\mathrm{II}}<1$.

Delays incurred in this phase include waiting time $w_{\mathrm{II}}$ and completion time $z_{\mathrm{II}}$. We note that the completion time $z_{\mathrm{II}}$ is not the same as the transmission time $\beta_{e}$. The former should take into account cases in which the transmission of a packet is interrupted by a coverage outage, so that the time taken to complete the transmission of a packet is prolonged by random coverage outages. The sojourn time in Phase II is $s_{\mathrm{II}}=w_{\mathrm{II}}+z_{\mathrm{II}}$.

### 3.2. Analysis of Queueing Parameters

**Figure 3:**
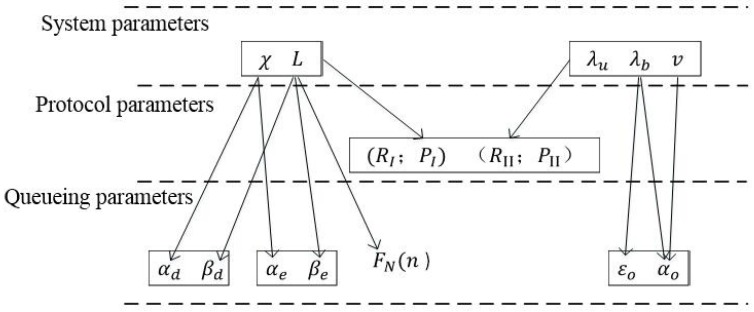
Relationships among system parameters, protocol parameters and queueing parameters.

So far, we have introduced the seven RVs that characterize our queueing model: $\alpha_{d}$, $\beta_{d}$, $\alpha_{e}$, $\beta_{e}$, $\alpha_{o}$, $\beta_{o}$ and *N*. We will subsequently show how these RVs are related to the various system and protocol parameters introduced in Section 2. A summary of the relationships among various parameters is illustrated in Figure 3.

#### 3.2.1. Assumptions

To facilitate a tractable analysis, we assume that $\alpha_{e}$ and $\alpha_{o}$ follow exponential distributions. In other words, Poisson arrivals are assumed for the effective traffic and the coverage outage processes. The Poisson assumption of $\alpha_{e}$ is a common practice in traffic engineering. The Poisson assumption of $\alpha_{o}$ is natural with PPP distributed BSs, as will be explained later in Section 3.2.2. We note that no particular distribution is assumed for $\alpha_{d}$ to justify the Poisson assumption of $\alpha_{e}$.

Because our system model assumes a fixed packet size and a constant broadcast rate, we have a deterministic $\beta_{d}\equiv L/R_{I}$. Our framework makes no particular assumptions on $\beta_{e}$ and $\beta_{o}$, i.e., both can follow general distributions. This gives our model the flexibility to represent and differentiate a wide range of practical systems. By varying the distributions of $\beta_{e}$, we can account for different policies and behaviors of open access small cells. Similarly, by varying the distributions of $\beta_{o}$, we can account for different user mobility patterns.

It is easy to see that the mean inter-arrival times of the original and effective traffic are both given by:

(11) $${\bar{\alpha }}_{d}={\bar{\alpha }}_{e}=L/C.$$

Moreover, the mean transmission times of the original and effective traffic are given by:

(12) $${\bar{\beta }}_{d}=L/R_{I}$$

and:

(13) $${\bar{\beta }}_{e}=L/R_{\mathrm{II}}$$

respectively.

#### 3.2.2. The Coverage Outage Process

The coverage outage process is fully characterized by RVs $\alpha_{o}$ and $\beta_{o}$. Here, we will show how their mean values ${\bar{\alpha }}_{o}$ and ${\bar{\beta }}_{o}$ are inherently related to the system parameters.

**Figure 4:**
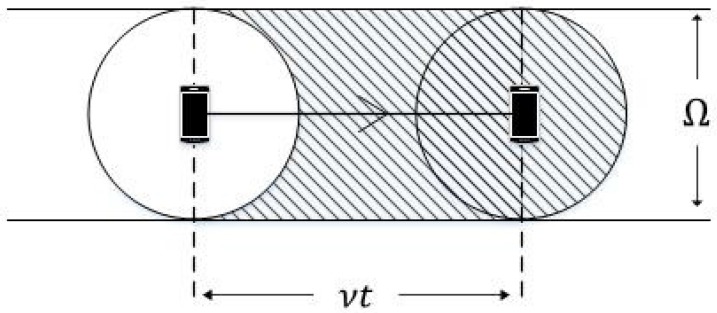
Coverage sensing area of a mobile user.

Let us first consider ${\bar{\alpha }}_{o}$. As shown in Figure 4, we assume that each user has a coverage sensing area represented by a circle, the diameter of which is given by Ω. When a user moves with speed *v* for a short period of time *t*, the movement trace can be regarded as a straight line. The sensing area covered by the mobile user during *t* is vtΩ, and new BSs may appear within this area. We assume that the user will attempt to handover to a newly appearing BS in the coverage sensing area, and an outage event occurs during a handover. Therefore, the rate of outage arrival is the same as the rate of BS arrival in the coverage sensing area. Because BSs follow a PPP distribution on the plane, it follows that:

(14) $${\bar{\alpha }}_{o}=\frac{1}{\lambda_{b}v\Omega }.$$

Now, we consider ${\bar{\beta }}_{o}$. As mentioned previously, we have $\varepsilon_{o}={\bar{\beta }}_{o}/{\bar{\alpha }}_{o}$; this parameter can be understood as the fraction of time that a user falls out of coverage. Parameter $\varepsilon_{o}$ depends on both the spatial coverage of the uplink and multi-user competition for access. We can write $\varepsilon_{o}=1-p_{c}p_{a}$, where $p_{c}$ is the probability that a user falls within coverage, and $p_{a}$ is the probability that the user is granted access among multiple users within the same cell. Coverage areas are defined as areas in which a receiver can receive a data rate higher than $R_{\mathrm{II}}$ in the presence of inter-cell interference. Because only one user is active in transmission in a cell, based on the interference models described in Section 2.2, we have $p_{c}={\tilde{F}}_{\chi }\left(2^{R_{\mathrm{II}}}-1;\lambda_{b},P_{II}\right)$, where function ${\tilde{F}}_{\chi }\left(x\right)$ is the interference complementary cumulative distribution function (CCDF) defined in Equation (6). Moreover, because all users have equal access to the BS, the multi-user access results in $p_{a}=\lambda_{b}/\lambda_{u}$ in an average sense (we assume that $\lambda_{u}>\lambda_{b}$ always holds). It follows that:

(15) $$\varepsilon_{o}=1-\frac{\lambda_{b}}{\lambda_{u}}{\tilde{F}}_{\chi }\left(2^{R_{\mathrm{II}}}-1;\lambda_{b},P_{II}\right).$$

#### 3.2.3. Number of Packet Copies *N*

All original traffic is broadcast in Phase I from its user with identical power $P_{I}$ and broadcast rate $R_{I}$. The broadcast is slotted with slot length $L/R_{I}$, where *L* is the fixed packet length. In each slot, the broadcasting users are called active users, while the rest are called inactive users. The time fraction that a user is active in broadcasting equals $\varepsilon_{d}={\bar{\beta }}_{d}/{\bar{\alpha }}_{d}$. The density of active users is therefore given by:

(16) $$\lambda_{a}=\lambda_{u}\varepsilon_{d}$$

and the density of inactive users is $\lambda_{w}=\lambda_{u}-\lambda_{a}$.

We assume that each inactive user is associated with the nearest active user and listens to its broadcast signal. Let *M* denote the number of associated inactive user per active user; the PMF of *M* is given by [51]:

(17) $$f_{M}\left(n\right)=\frac{3.5^{3.5}}{\Gamma (3.5)n!}\frac{\Gamma \left(n+3.5\right){\left(\frac{\lambda_{w}}{\lambda_{a}}\right)}^{n}}{{\left(\frac{\lambda_{w}}{\lambda_{a}}+3.5\right)}^{n+3.5}}$$

where Γ(·) denotes the Gammafunction and (·)! denotes factorial.

For each active user, the number of inactive users that can successfully receive its broadcast in each time slot is an RV denoted by $N^{\prime }$. The number of copies of a packet after a broadcast is denoted by *N*, and we have $N=N^{\prime }+1$. An inactive user can successfully receive a packet only if it can receive Phase I broadcasting with an SINR higher than $\chi =2^{R_{I}}-1$. The probability of successful packet reception can be calculated as $F_{\gamma }\left(\chi ;\lambda_{a},P_{I}\right)$. Because the transmitter is assumed to follow an ergodic PPP process, the SINR can be treated as spatially ergodic. It follows that $N^{\prime }∼B\left(M,F_{\gamma }\left(\chi ;\lambda_{a},P_{I}\right)\right)$, i.e., $N^{\prime }$ follows a binomial distribution with parameters *M* and $p=F_{\gamma }\left(\chi ;\lambda_{a},P_{I}\right)$. Since *M* is an RV, the PMF of $N^{\prime }$ can be obtained by taking the expectation over *M*, i.e.,

(18) $$f_{N^{\prime }}\left(n\right)=\sum_{m=0}^{\infty }f_{M}\left(n\right)C_{m}^{n}p^{n}{\left(1-p\right)}^{m-1}\;\;\;\left(n\geq 0\right)$$

where $C_{m}^{n}=m!/n!$. Finally, the PMF of the random number of copies of a packet in the system is given by:

(19) $$f_{N}\left(n\right)=f_{N^{\prime }}\left(n-1\right)\;\;\;\;\;\;\left(n\geq 1\right).$$

## 4. Capacity Limits and Delay Analysis

### 4.1. Capacity Limits

Consider the priority queue in the deliver phase; the combined load of two classes of traffic is given by:

(20) $$\varepsilon_{\mathrm{II}}=\varepsilon_{e}+\varepsilon_{o}$$

where $\varepsilon_{e}=C/R_{\mathrm{II}}$ and $\varepsilon_{o}$ is defined in Equation (15). A stable queue requires $\varepsilon_{\mathrm{II}}<1$; it follows that:

(21) $$C<\frac{\lambda_{b}}{\lambda_{u}}\frac{R_{\mathrm{II}}}{F_{\gamma }\left(2^{R_{\mathrm{II}}-1};\lambda_{b},P_{\mathrm{II}}\right)}.$$

Given BS density $\lambda_{b}$ and power input $P_{\mathrm{II}}$, the capacity *C* can be optimized over $R_{\mathrm{II}}$, i.e.,

(22) $$C^{*}\left(\lambda_{b},P_{\mathrm{II}}\right)=\max_{R_{\mathrm{II}}}\frac{\lambda_{b}}{\lambda_{u}}\frac{R_{\mathrm{II}}}{F_{\gamma }\left(2^{R_{\mathrm{II}}-1};\lambda_{b},P_{\mathrm{II}}\right)}.$$

Numerical results show that *C* appears convex over $R_{\mathrm{II}}$ under various parameter settings. Therefore, $C^{*}\left(\lambda_{b},P_{\mathrm{II}}\right)$ can be calculated by effective numerical methods. Furthermore, it is easy to see that $C^{*}$ is a monotonically-increasing function of $P_{\mathrm{II}}$. From a theoretical point of view, we are interested in the fundamental capacity limit $C_{\lim}$ defined as:

(23) $$C_{\lim}=\lim_{P_{\mathrm{II}}\to \infty }C^{*}\left(\lambda_{b},P_{\mathrm{II}}\right).$$

Substitute Equations (9) and (22) into Equation (23), we get:

(24) $$C_{\lim}=\max_{x}\frac{\lambda_{b}}{\lambda_{u}}\frac{x}{1+\sqrt{2^{x}-1}\arctan\left(1/\sqrt{2^{x}-1}\right)}.$$

**Figure 5:**
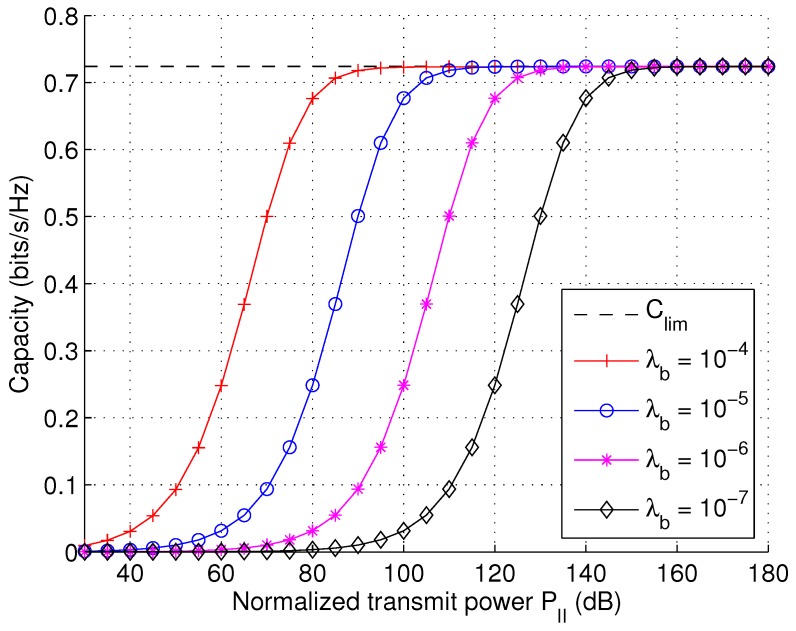
Maximum capacity per device as a function of transmit power $P_{\mathrm{II}}$ with varying infrastructure density $\lambda_{b}$ ($\lambda_{b}/\lambda_{u}$ =1).

It can be shown that $C_{\lim}$ is a convex function of *x*. Numerical evaluation can be performed to give $C_{\lim}=0.7239$ $\lambda_{b}/\lambda_{u}$ bits/s/Hz, which shows a constant scaling with $\lambda_{b}/\lambda_{u}$. We note that our conclusion conforms with the conclusions obtained via scaling law analysis [28], which predicts that the capacity can grow linearly with $\lambda_{b}/\lambda_{u}$. Our model refines the result by obtaining the exact scaling constant as 0.7239. In Figure 5, the optimal capacity $C^{*}$ is illustrated as a function of $P_{\mathrm{II}}$ with varying $\lambda_{b}$ based on Equation (22). It is observed that $C^{*}$ increases initially with increasing $P_{\mathrm{II}}$ or $\lambda_{b}$, but eventually reaches the upper bound $C_{\lim}$.

### 4.2. Delay Distributions

This subsection aims to obtain the exact distribution of the four delay parameters $w_{I}$, $z_{I}$, $w_{\mathrm{II}}$ and $z_{\mathrm{II}}$, from which the total delay can be obtained as:

(25) $$D=w_{I}+z_{I}+w_{\mathrm{II}}+z_{\mathrm{II}}$$

The PDF of *D* can be numerically calculated as the convolution of the PDFs of each component.

#### 4.2.1. Phase I Delays $w_{I}$ and $z_{I}$

Because we have assumed a fixed packet size and a fixed broadcast rate $R_{I}$, it is obvious that:

(26) $$z_{I}\equiv {\bar{\beta }}_{d}\equiv \frac{L}{R_{I}}.$$

The queueing process in Phase I forms a G/D/1, queue and the exact distribution of $w_{I}$ is generally unavailable. In the special case that $\alpha_{d}$ follows exponential distributions, the queueing process in Phase I becomes an M/D/1queue, and we have [52]:

(27) $$F_{w_{I}}\left(t\right)=\left(1-{\bar{\alpha }}_{d}\right)\sum_{n=0}^{K}\frac{\alpha_{d}{\left(n-t\right)}^{n}}{n!}e^{-\alpha_{d}\left(n-t\right)}$$

where $K=\left\lfloor t\right\rfloor$ is the largest integer smaller than *t*. The average waiting time is given by [52]:

(28) $${\bar{w}}_{I}=\frac{1}{2}\frac{\varepsilon_{d}}{1-\varepsilon_{d}}{\bar{\beta }}_{d}.$$

These results of an M/D/1 queue can serve as a reasonable estimate for the actual delay of the G/D/1 queue under practical settings. We note that under practical settings, the Phase II delays are much greater than Phase I delays, i.e., $w_{\mathrm{II}}+z_{\mathrm{II}}>>w_{I}+z_{I}$. Therefore, our subsequent focus is on obtaining the exact distributions of $w_{\mathrm{II}}$ and $z_{\mathrm{II}}$.

#### 4.2.2. Phase II Completion Time $z_{\mathrm{II}}$

In Phase II, we have an M/G/1 priority queue with two classes of traffic. The first-class of traffic is coverage outage, while the second-class of traffic is effective traffic. We are interested in the completion time of the second class of traffic. The Laplace transform of $z_{\mathrm{II}}$ is given by [52]:

(29) $$L_{z_{\mathrm{II}}}\left(s\right)=L_{\beta_{e}}\left[K\left(s\right)\right]$$

where $L_{\beta_{e}}\left(\cdot \right)$ is the Laplace transform of $\beta_{e}$ and:

(30) $$K\left(s\right)=s+\frac{1-G\left(s\right)}{{\bar{\alpha }}_{o}}.$$

Here, G(s) is the solution with the smallest absolute value that satisfies the following equation:

(31) $$x-L_{\beta_{o}}\left(s+\frac{1-x}{{\bar{\alpha }}_{o}}\right)=0$$

where $L_{\beta_{o}}\left(\cdot \right)$ is the Laplace transform of $\beta_{o}$. From (29)–(31), the Laplace transform $L_{z_{\mathrm{II}}}\left(s\right)$ can be obtained. The exact PDF of $z_{\mathrm{II}}$ can then be numerically calculated using standard methods of Laplace inversion. Finally, the first and second moment of $z_{\mathrm{II}}$ can be evaluated analytically as [52]:

(32) $${\bar{z}}_{\mathrm{II}}=\frac{\beta_{e}}{1-\varepsilon_{o}}$$

and:

(33) $${\hat{z}}_{\mathrm{II}}=\frac{{\hat{\beta }}_{o}}{{\left(1-\varepsilon_{o}\right)}^{2}}+{\hat{\beta }}_{o}\frac{{\bar{\beta }}_{e}}{{\bar{\beta }}_{o}}\frac{{\hat{\varepsilon }}_{o}}{{\left(1-\varepsilon_{o}\right)}^{3}}$$

respectively.

#### 4.2.3. Discussions on $\beta_{o}$

We have so far assumed a general distribution for the outage duration $\beta_{o}$. This distribution affects the solution of Equation (31). We will subsequently discuss two special distributions for $\beta_{o}$.

The first distribution to consider is the exponential distribution. This memoryless distribution is a natural choice for $\beta_{o}$ when small cell BSs are randomly located as a PPP and users have coverage-independent mobility patterns. Given $\beta_{o}∼\exp\left(1/{\bar{\beta }}_{o}\right)$, its Laplace transform can be evaluated as:

(34) $$L_{\beta_{o}}^{E}\left(s\right)=\frac{1}{1+s{\bar{\beta }}_{o}}.$$

It follows that Equation (31) can be solved explicitly to give:

(35) $$G\left(s\right)=\frac{\left(1+\varepsilon_{o}+s{\bar{\beta }}_{o}\right)-\sqrt{{\left(1+\varepsilon_{o}+s{\bar{\beta }}_{o}\right)}^{2}-4\varepsilon_{o}}}{2\varepsilon_{o}}$$

Another useful distribution we consider is the Gamma distribution. The Gamma distribution can provide more flexibility when characterizing $\beta_{o}$ for a variety of practical scenarios. Given $\beta_{o}∼\Gamma \left(k,\theta \right)$, the PDF of $\beta_{o}$ is given by:

(36) $$f_{\beta_{o}}\left(t\right)=\frac{1}{\theta^{k}}\frac{1}{\Gamma \left(k\right)}t^{k-1}e^{-\frac{t}{\theta }}$$

where *k* and *θ* are the shape and scale parameters, respectively. Under the Gamma distribution, the Laplace transform of $\beta_{o}$ is given by:

(37) $$L_{\beta_{o}}^{G}\left(s\right)={\left(1+\theta s\right)}^{-k}$$

It follows that when *k* is an integer or a rational fraction, Equation (31) yields a polynomial form. Therefore, function G(s) can be easily solved using existing root-finding algorithms for polynomials.

#### 4.2.4. Phase II Waiting Time $w_{\mathrm{II}}$

The waiting time $w_{\mathrm{II}}$ of a packet depends on its traffic type, i.e., the number of packet copies in the system. We denote the waiting time of a type-*n* traffic as $w_{\mathrm{II}}^{n}$. Let us first consider $w_{\mathrm{II}}^{1}$, whose Laplace transform is given by [52]:

(38) $$L_{w_{\mathrm{II}}^{1}}\left(s\right)=\left(1-\varepsilon_{\mathrm{II}}\right){\bar{\alpha }}_{e}\frac{K\left(s\right)}{L_{\beta_{e}}\left[K\left(s\right)\right]+{\bar{\alpha }}_{e}s-1}$$

where K(s) is defined in Equation (30). It is possible that the packet arrives to see an empty buffer. Therefore, the CDF function has a non-zero value at 0+, which is given by [52]:

(39) $$F_{w_{\mathrm{II}}^{1}}\left(t=0+\right)=1-\varepsilon_{\mathrm{II}}.$$

Clearly, the CDF of the virtual waiting time depends on the characteristics of the effective traffic and coverage outage process.

**Figure 6:**
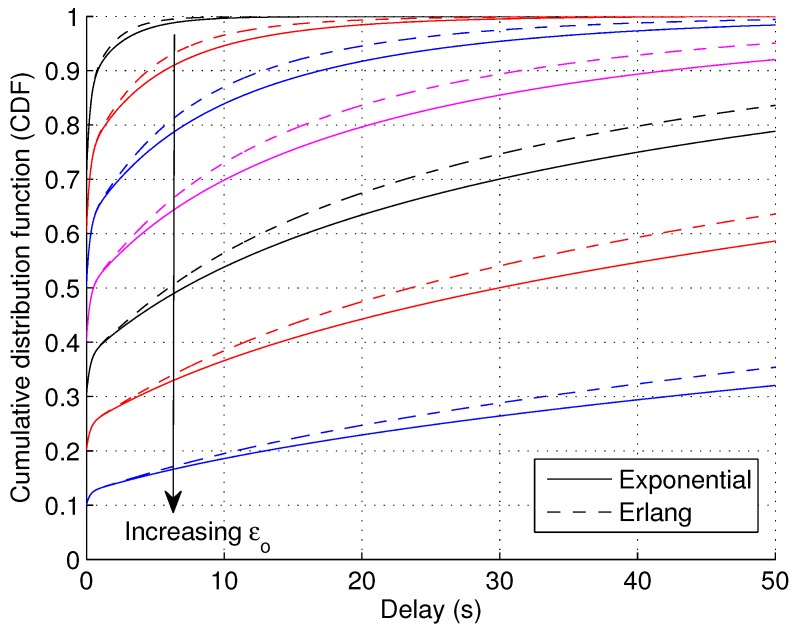
CDF of waiting time $w_{\mathrm{II}}$ with varying coverage outage fraction $\varepsilon_{o}$ when the coverage outage duration $\beta_{o}$ follows the exponential and Gamma distribution ($\varepsilon_{o}$ increases from 0.1–0.7 with steps of 0.1, *k* = 2, *N* = 1, ${\bar{\alpha }}_{o}$ = 20, $\varepsilon_{e}$ = 0.2, ${\bar{\alpha }}_{e}$ = 1).

In Figure 6, the CDF of $w_{\mathrm{II}}^{1}$ is illustrated with varying values of $\varepsilon_{o}$, which denotes the fraction of areas without coverage. Similar to the definition of the well-known “outage capacity” in fading channels, we can define “outage delay” as the delay that grantees certain outage. For example, a 10% outage delay is the delay $t_{10}$ that satisfies $F_{w_{\mathrm{II}}}\left(t_{10}\right)=0.9$. From Figure 6, a nonlinear relationship is observed between $\varepsilon_{o}$ and outage delays. Taking the 10% outage delay for example, when $\varepsilon_{o}$ takes values of 0.1, 0.2, 0.3 and 0.4, the corresponding 10% outage delay is roughly 1 s, 7 s, 17 s and 43 s, respectively. Therefore, the delay performance degrades quickly with increasing coverage outage.

Another aspect we investigate in Figure 6 is how the CDF of $w_{\mathrm{II}}^{1}$ is influenced by different distributions of $\beta_{o}$. Two types of distributions are compared: one is the exponential distribution, the other the Gamma distribution with *k* = 2, which is also an Erlang distribution. The former distribution corresponds to a purely random network, while the latter can represent networks that are planned with certain regularities. For the purpose of fair comparison, the two types of distributions are set to have the same mean ${\bar{\beta }}_{o}$. It is observed that the Erlang distribution gives slightly better performance than the exponential distribution. From this, we postulate that the delay performance will improve if the small cell network is not entirely random, but exhibits certain regularities.

Now, consider the waiting time of a type-*n* traffic packet. Because there are now *n* copies undergoing independent queueing processes, the actual waiting time $w_{\mathrm{II}}^{n}$ is the minimum of the *n* queues. The delay CDF of a type-*n* traffic packet can then be evaluated as:

(40) $$F_{w_{\mathrm{II}}^{n}}\left(t;n\right)=1-{\left[1-F_{w_{\mathrm{II}}^{1}}\left(t\right)\right]}^{n}.$$

Further consider *n* as an RV denoted by *N* and apply the law of total probability; the CDF of the waiting time of an arbitrary packet is given by:

(41) $$F_{w_{\mathrm{II}}}\left(t\right)=\sum_{1}^{\infty }f_{N}\left(n\right)\left(1-{\left[1-F_{w_{\mathrm{II}}^{1}}\left(t\right)\right]}^{N}\right)$$

where $f_{N}\left(n\right)$ is the PDF of *N* given by (19).

**Figure 7:**
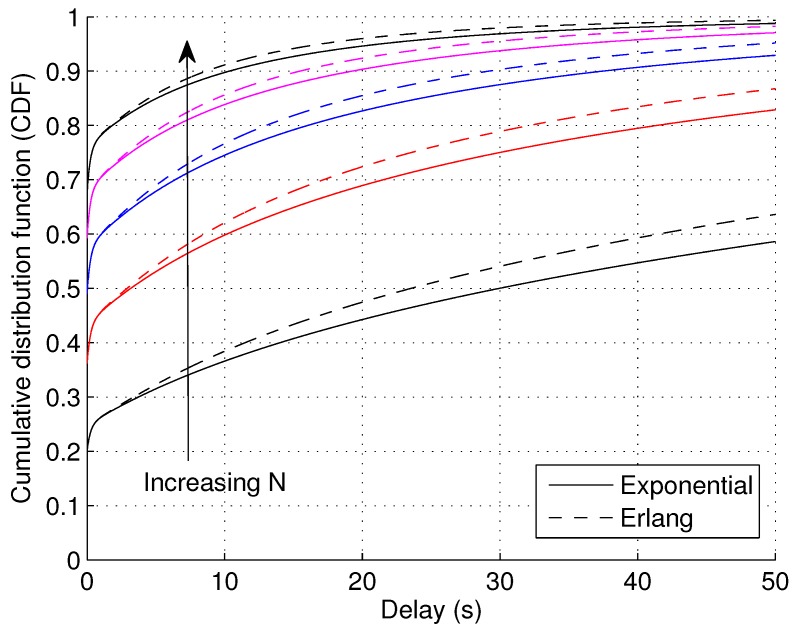
CDF of waiting time $w_{\mathrm{II}}$ with varying number of collaborating devices *N* when the service outage duration $\beta_{o}$ follows the exponential and Gamma distribution (*N* increases from 1–5 with steps of one, *k* = 2, $\varepsilon_{o}$ = 0.6, ${\bar{\alpha }}_{o}$ = 20, $\varepsilon_{e}$ = 0.2, ${\bar{\alpha }}_{e}$ = 1).

In Figure 7, the CDF of $w_{\mathrm{II}}^{N}$ is illustrated with varying values of *N* according to Equation (40). User collaboration is shown to be effective in reducing delays. Compare Figure 7 with Figure 6; we observe that the performance given by N=5 and $\varepsilon_{o}=0.6$ is comparable to the performance given by N=1 and $\varepsilon_{o}=0.2$. In other words, if a packet is successfully broadcast to four other users, the coverage requirement can be relaxed about two times in this case ((1−0.2)÷(1−0.6)=2). On the other hand, Figure 7 also shows that the benefits of increasing *N* gradually diminishes as *N* goes large.

## 5. Rate and Power Optimization

In the previous section, we have established the delay distribution subject to protocol parameters and system parameters. From the practical perspective of system design and optimization, it is desirable to understand how the protocol parameters ($R_{I}$, $R_{\mathrm{II}}$, $P_{I}$ and $P_{\mathrm{II}}$) can be properly chosen to give an optimized capacity-delay performance. Without loss of generality, our subsequent analysis is restricted to the case where both $\beta_{e}$ and $\beta_{o}$ follow exponential distributions.

### 5.1. Heuristic Optimization of $R_{I}$

Under natural conditions, the waiting time $w_{\mathrm{II}}$ dominates the delay. Therefore, the primary target of delay minimization is to minimize $w_{\mathrm{II}}$. According to Figure 7, increasing the number of packet copies is shown to be very effective in reducing delays. Therefore, a simple heuristics for the optimization of $R_{I}$ is to maximize the mean number of packet copies $\bar{N}$. It turns out a simple closed-form estimate exists to give $\bar{N}=\bar{J}q$, where:

(42) $$\bar{J}=\left(\lambda_{u}-\lambda_{a}\right)/\lambda_{a}=R_{I}/C-1$$

denotes the ratio of inactive users and active users in Phase I, $\lambda_{a}=\lambda_{u}C/R_{I}$, and:

(43) $$q=F_{\gamma }\left(2^{R_{I}}-1;\lambda_{a},P_{I}\right)$$

denotes the probability that an inactive user can successfully receive a packet. Because increasing $R_{I}$ will increase $\bar{J}$, but reduce *q*, such a tension requires an optimization over $R_{I}$. The optimization problem of $R_{I}$ can be formulated as follows: given *C*, $\lambda_{u}$ and $P_{I}$,

(44) $$R_{I}^{*}=\arg\max_{x}\left(\frac{x}{C}-1\right)F_{\gamma }\left(2^{x}-1;\frac{\lambda_{u}C}{x},P_{I}\right)\;\;\;\;\;\;x>0.$$

**Figure 8:**
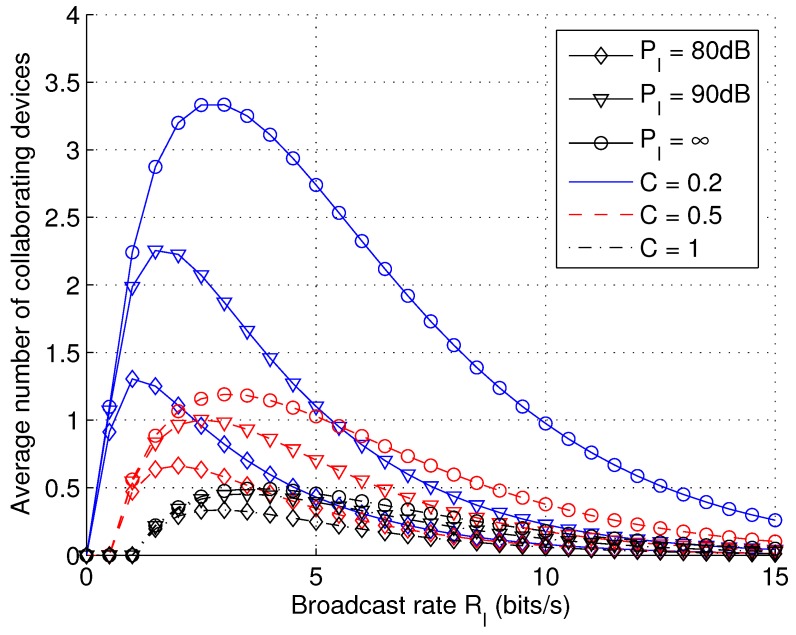
The average number of collaborating devices $\bar{N}$ as a function of broadcast rate $R_{I}$ with varying transmit power $P_{I}$ and capacity demand *C* ($\lambda_{u}$ = $10^{-4}$).

Figure 8 shows $\bar{N}$ as a function of $R_{I}$. The above optimization problem is shown to have a simple structure with a single peak value, which can be easily obtained via numerical methods.

### 5.2. Heuristic Optimization of $R_{\mathrm{II}}$

The total delay is dominated by the waiting time $w_{\mathrm{II}}$, which depends largely on the waiting time of Type-1 traffic $w_{\mathrm{II}}^{1}$. A simple heuristic to optimize $R_{\mathrm{II}}$ is therefore to minimize the mean of $w_{\mathrm{II}}^{1}$ given by:

(45) $${\bar{w}}_{\mathrm{II}}^{1}=\frac{1}{2\left(1-\varepsilon_{o}\right)\left(1-\varepsilon_{\mathrm{II}}\right)}\left(\frac{2{\bar{\beta }}_{e}^{2}}{{\bar{\alpha }}_{e}}+\frac{2{\bar{\beta }}_{o}^{2}}{{\bar{\alpha }}_{o}}\right)$$

in the case that $\beta_{e}$ and $\beta_{o}$ both follow exponential distributions. Increasing $R_{\mathrm{II}}$ will reduce the Phase II transmission time (once in coverage), but at the cost of reduced probability to fall within coverage. This tension leads to an optimization problem as follows: given *C*, $P_{\mathrm{II}}$, *L*, *v* and $\lambda_{b}$,

(46) $$R_{\mathrm{II}}^{*}=\arg\max_{R_{\mathrm{II}}}{\bar{w}}_{\mathrm{II}}\;\;\;\;\;$$

**Figure 9:**
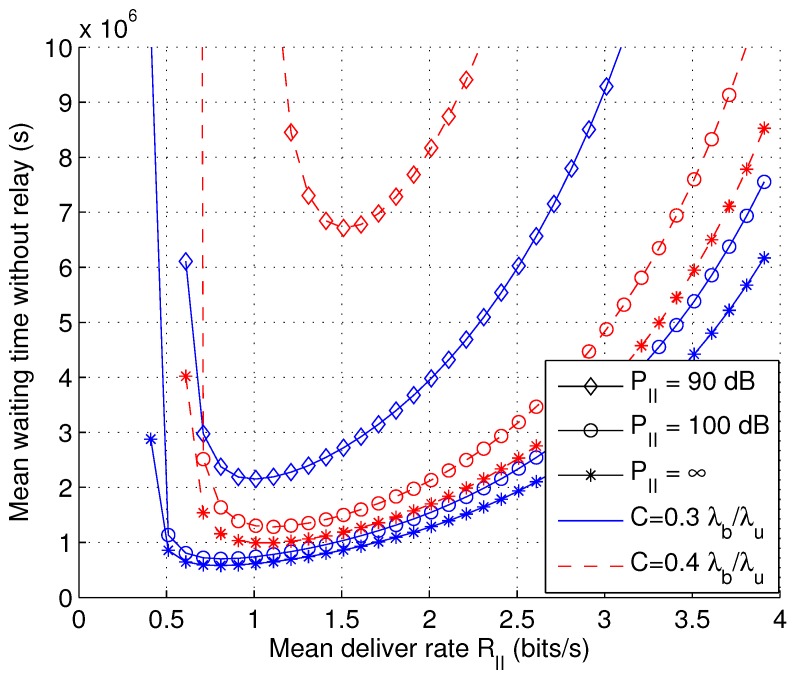
The mean waiting time ${\bar{w}}_{\mathrm{II}}^{1}$ as a function of average delivery rate $R_{\mathrm{II}}$ with varying transmit power $P_{\mathrm{II}}$ and capacity demand *C* ($\lambda_{b}$ = $10^{-6}$, *L* = 1, *v* = 1).

In Figure 9, the mean waiting time ${\bar{w}}_{\mathrm{II}}^{1}$ is shown as a function of average delivery rate $R_{\mathrm{II}}$ with varying transmit power $P_{\mathrm{II}}$ and capacity demand *C*. The objective function appears to be convex, and the optimal value can be easily obtained via numerical methods.

### 5.3. Heuristic Optimization of Power $P_{I}$

Unlike the optimizations over $R_{I}$ and $R_{\mathrm{II}}$ that aim to balance between conflicting effects, increasing $P_{I}$ is always beneficial, but with diminishing returns in terms of capacity and delay. Our heuristic approach to the optimization of $P_{I}$ is based on the following idea: when $P_{I}$ reaches a threshold, further increasing $P_{I}$ is not helpful as Phase I broadcasting becomes interference limited. Therefore, we want to have the minimum $P_{I}$ that can achieve ϕ percent of the best performance given by $P_{I}\to \infty$. As mentioned previously, the probability for an inactive user to successfully receive a packet is $F_{\gamma }\left(\chi \right)$. This can be used as a convenient performance indicator of the broadcasting performance.

The optimization of $P_{I}$ can now be formulated as follows:

(47) $$P_{I}^{*}=\arg\min_{P}\frac{F_{\gamma }^{*}\left(\chi ;\lambda_{a},P\right)}{F_{\gamma }^{\lim}\left(\chi ;\lambda_{a},\infty \right)}⩾\phi ,\;\;\phi \in \left(0,1\right)$$

where $\chi =2^{R_{I}}-1$, $\lambda_{a}=\lambda_{u}\varepsilon_{d}$ and functions $F_{\gamma }^{*}\left(\cdot \right)$ and $F_{\gamma }^{\lim}\left(\cdot \right)$ are defined in Equations (8) and (9), respectively.

At relatively high values of $P_{I}$, the *Q*-function appearing in Equation (8) can be well approximated by a lower bound given by:

(48) $$Q\left(x\right)⪆\frac{x^{2}}{\sqrt{2\pi }\left(1+x^{2}\right)}e^{-x^{2}/2}.$$

Substituting Equations (8), (9) and (48) into Equation (47), we get:

(49) $$P_{I}^{*}\approx \frac{\phi }{1-\phi }\frac{2\chi }{{\left(\pi \lambda_{a}\right)}^{2}{\left[1+\sqrt{\chi }\arctan\left(\sqrt{\chi }\right)\right]}^{2}}.$$

Equation (49) gives a closed-form formula to directly calculate $P_{I}^{*}$ from system parameters.

### 5.4. Heuristic Optimization of Power $P_{\mathrm{II}}$

The idea for the optimization of $P_{\mathrm{II}}$ is similar to that of $P_{I}$, only that the objective function is now $C_{\lim}$. As shown in Figure 5, increasing $P_{I}$ is always beneficial to the capacity until the capacity approaches a constant limit. The optimization problem can be formulated as:

(50) $$P_{\mathrm{II}}^{*}=\arg\min_{P}\frac{C^{*}\left(\lambda_{b},P\right)}{C_{\lim}}\geq \phi \;\;\;\left(0<\phi <1\right)$$

where $C^{*}\left(\lambda_{b},P\right)$ and $C_{\lim}$ are defined in Equations (22) and (24), respectively. Obviously, given $\lambda_{b}$, $P_{\mathrm{II}}^{*}$ can be easily obtained from Figure 5 by drawing a horizontal line at $C=\phi *C_{\lim}=0.7329\phi$ to intersect with the various curves.

## 6. Numerical Results and Discussions

In this section, numerical results are shown to illustrate the capacity-delay trade-off with varying system parameters. We aim to shed light on the following questions: What is the trade-off between capacity and delay? How does this trade-off change with different system parameters? We note that two different metrics for the delay performance can be considered: the mean delay and the outage delay. Due to page limits, our discussions are limited to the mean delay.

The procedure of our numerical evaluation is as follows: (1) given system parameters (*C*, *L*, $\lambda_{u}$, $\lambda_{b}$, and *v*) and power parameters ($P_{I}$ and $P_{\mathrm{II}}$), calculate the optimal rate parameter $R_{I}$ and $R_{\mathrm{II}}$ according to Equations (44) and (46), respectively; (2) given all of the above parameters, calculate the PDFs of $w_{\mathrm{II}}$ and $z_{\mathrm{II}}$ based on Section 4; (3) calculate the PDF of the accumulated delay *D* and evaluate its mean value $\bar{D}$. Without loss of generality, we set $\lambda_{b}=10^{-5}$ and Ω=100 in all cases.

**Figure 10:**
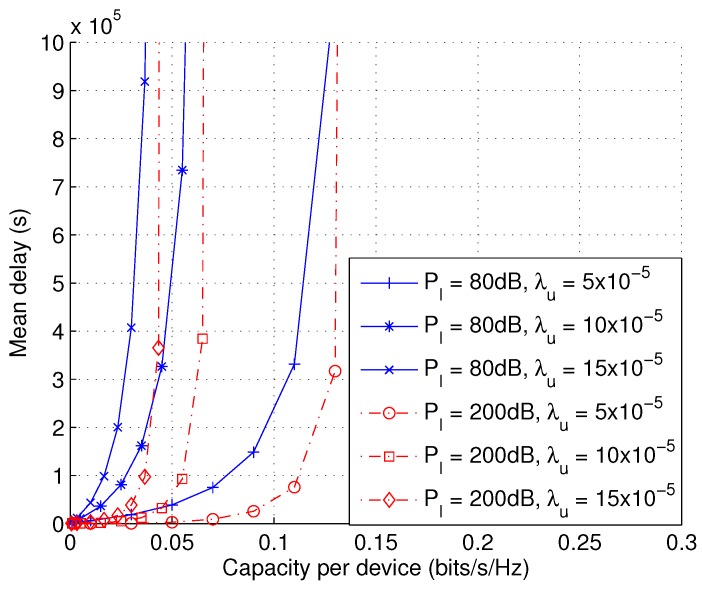
Mean delay $\bar{D}$ as a function of capacity per device *C* with varying transmit power $P_{I}$ and user density $\lambda_{u}$ (*L* = 1, $P_{\mathrm{II}}=\infty$, $\lambda_{b}$ = $10^{-5}$, *v* = 1).

Figure 10 shows the impact of user density $\lambda_{u}$ and power $P_{I}$ on the capacity-delay trade-off. The trade-off is shown to be insensitive to the user density. This is because the capacity limit scales with $0.7239\lambda_{b}/\lambda_{u}$. When capacity approaches this limit, the delay shows an exponential growth to infinity. The value of $P_{I}$ is also shown to have a significant impact on the delay performance. The case of $P_{I}=200$ dB represents the extreme case of infinite power. The capacity-delay trade-off at $P_{I}=200$ dB indicates the performance upper bound we can get from user collaboration.

**Figure 11:**
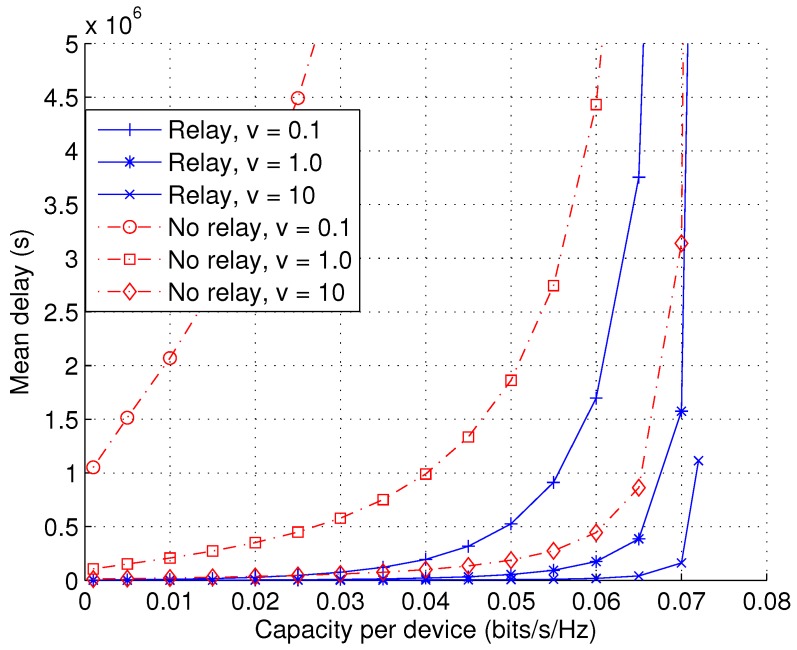
Mean delay $\bar{D}$ as a function of capacity per device *C* with varying user mobile speed *v* (*L* = 1, $P_{I}=\infty$, $P_{\mathrm{II}}=\infty$, $\lambda_{b}$ = $10^{-5}$, arbitrary $\lambda_{u}$).

Figure 11 shows the impact of user speed *v* on the capacity-delay trade-off, for cases with and without user collaboration. We set $P_{I}$ and $P_{\mathrm{II}}$ to very large values to shed light on to the fundamental performance limits. The trade-off is shown to be sensitive to the speed. For a ten-fold increase of the speed, the delay is shown to reduce by about 90%. In other words, an inversely proportional relationship is observed between speed and mean delay. The benefit of user collaboration (i.e., relay) is shown to be significant, especially when the movement speed is low. This suggests that in practice, allowing D2D communications between low speed and high speed users will effectively reduce the delays of low speed users.

**Figure 12:**
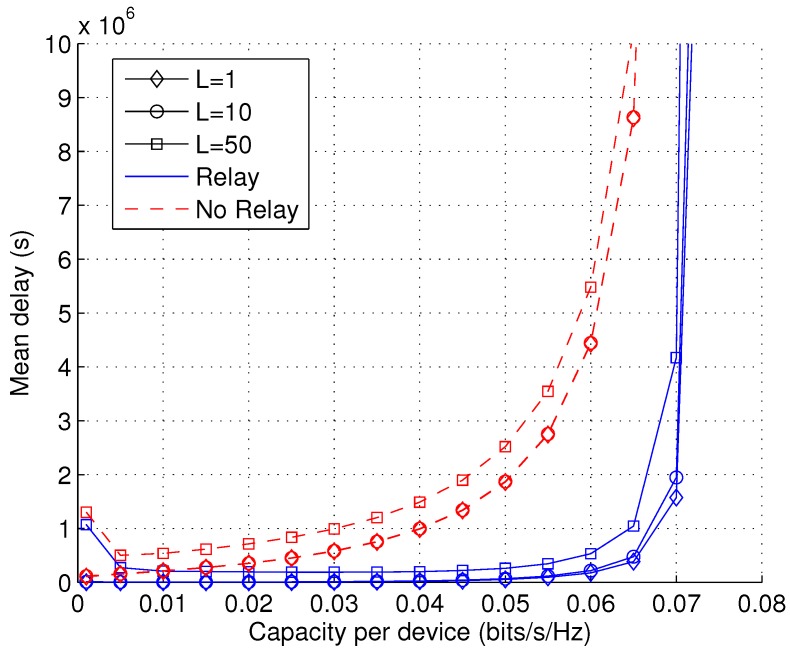
Mean delay $\bar{D}$ as a function of capacity per device *C* with varying packet size *L* ($P_{I}=\infty$, $P_{\mathrm{II}}=\infty$, $\lambda_{b}$ = $10^{-5}$, arbitrary $\lambda_{u}$, *v* = 1).

Figure 12 shows the impact of packet size *L* on the capacity-delay trade-off. In practice, it is desirable to have a larger packet size to reduce overhead. However, it is observed that increasing *L* leads to slightly increased delays. This suggests that the packet size should also be optimized carefully in practice. It is interesting to see that the delay becomes larger when the value of *C* approaches zero. This is because the heuristic algorithms for optimizing protocol parameters are sub-optimal for very small values of *C*. This shows some limitations of the heuristic algorithm in Section 5.

**Figure 13:**
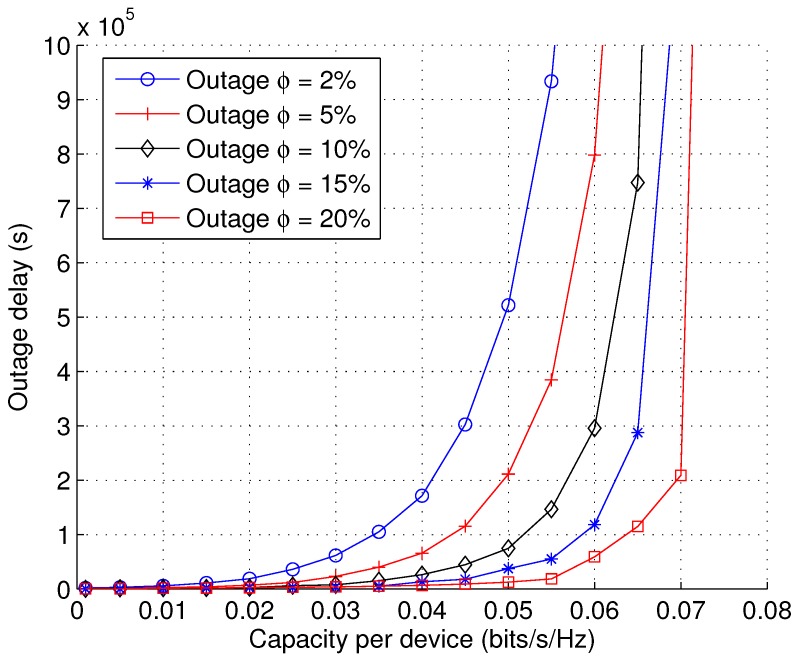
Outage delay D(ϕ) as a function of capacity per device *C* with varying outage threshold ϕ ($P_{I}=\infty$, $P_{\mathrm{II}}=\infty$, $\lambda_{b}$ = $10^{-5}$, arbitrary $\lambda_{u}$, L=1, *v* = 1).

While all of the above numerical results are based on the mean delay, it is also important to investigate the trade-off performance in terms of the outage delay. In practice, a small fraction of packets with large delays is allowed to be dropped by the queue; hence, the outage delay is particularly useful when the delay has long-tail distributions. Given a random delay *D* and its CDF $F_{D}\left(x\right)$, the outage delay $D_{o}\left(\phi \right)$ is defined as the delay value that gives $F_{D}\left(D_{o}\right)=1-\phi$, where ϕ is the outage threshold. In Figure 13, we show the capacity-delay trade-off based on outage delay. As expected, we see that the outage delay increases in an exponential fashion when the capacity per user approaches the limit. Moreover, it is observed that the delay reduces with increasing outage probability ϕ. Finally, we note that being able to pinpoint the delay distribution and study the outage delay performance is a key merit of the analytical framework proposed in this paper. Our analytical framework can potentially be extended beyond the scenario of cellular communications and applied to other networking paradigms, such as multi-hop sensor networks [53,54,55].

## 7. Conclusions

This paper has studied the uplink capacity-delay trade-off of large-scale hybrid wireless networks with a two-hop broadcast-and-forward relaying scheme. A queueing theoretic framework has been established to evaluate the exact distribution of the delays. The impacts of transmission rates, transmission power, user density, BSs density and packet size on the capacity-delay trade-off have been thoroughly investigated. Heuristic power and rate control algorithms have been proposed for performance optimization. Using a different and independent model, we reach the same conclusion with existing literature that per-user capacity scales with BS-user density ratio. However, our model is able to give an exact scaling coefficient as 0.7239 in the interference limiting scenario. Numerical results suggest that mobility and user collaboration are effective means to reduce the mean and outage packet delay.

## Figures and Tables

**Table 1:** List of the main parameters.

System Parameters		Protocol Parameters		Queueing Parameters	
*χ*	Packet arrival rate	$R_{I}$	Transmit rate in broadcast phase	$\alpha_{d}$	Arrival interval of original traffic
*L*	Packet size	$P_{I}$	Transmit power in broadcast phase	$\beta_{d}$	Transmission time of original traffic
$\lambda_{u}$	User density	$R_{II}$	Transmit rate in deliver phase	$\alpha_{e}$	Arrival interval of effective traffic
$\lambda_{b}$	BS density	$P_{II}$	Transmit power in deliver phase	$\beta_{e}$	Transmit time of effective traffic
*v*	User speed	$p_{c}$	Coverage probability	$\varepsilon_{e}$	Load of effective traffic
*C*	User capacity demand	$p_{a}$	Access probability	$\alpha_{o}$	Arrival interval of coverage outage
*η*	Path loss exponent	*N*	Number of collaborative users	$\beta_{o}$	Duration of coverage outage
ϕ	Probability of delay outage	$F_{N}\left(n\right)$	CDF of *N*	$\varepsilon_{o}$	Load of coverage outage

## References

[1] Cisco T. Cisco Visual Networking Index: Global Mobile Data Traffic Forecast Update 2015–2020 White Paper. http://www.cisco.com/c/en/us/solutions/collateral/service-provider/visual-networking-index-vni/mobile-white-paper-c11-520862.html.

[2] Hoydis J., Kobayashi M., Debbah M. (2011). Green small-cell networks. IEEE Veh. Technol. Mag..

[3] Doppler K., Rinne M., Wijting C., Ribeiro C.B., Hugl K. (2009). Device-to-device communication as an underlay to LTE-advanced networks. IEEE Commun. Mag..

[4] Fodor G., Dahlman E., Mildh G., Parkvall S., Reider N., Miklos G., Turanyi Z. (2012). Design aspects of network assisted device-to-device communications. IEEE Commun. Mag..

[5] Zhou S., Gong J., Zhou Z., Chen W., Niu Z. (2015). Green delivery: Proactive content caching and push with energy-harvesting-based small cells. IEEE Commun. Mag..

[6] Wang X., Chen M., Taleb T., Ksentini A., Leung V.C.M. (2014). Cache in the air: Exploiting content caching and delivery techniques for 5G systems. IEEE Commun. Mag..

[7] Zhao N., Liu X., Yu F.R., Li M., Leung V.C.M. (2016). Communications, caching, and computing oriented small cell networks with interference alignment. IEEE Commun. Mag..

[8] Tourani R., Misra S., Mick T. (2016). IC-MCN: An architecture for an information-centric mobile converged network. IEEE Commun. Mag..

[9] Liu D., Chen B., Yang C., Molisch A.F. (2016). Caching at the wireless edge: Design aspects, challenges, and future directions. IEEE Commun. Mag..

[10] Gupta P., Kumar P.R. (2000). The capacity of wireless networks. IEEE Trans. Inf. Theory.

[11] Buragohain C., Suri S., Tóth C.D., Zhou Y. Improved Throughput Bounds for Interference-aware Routing Inwireless Networks. Proceedings of the 13th Annual International Conference on Computing and Combinatorics.

[12] Dousse O., Franceschetti M., Thiran P. (2006). On the throughput scaling of wireless relay networks. IEEE Trans. Inf. Theory.

[13] Duarte-Melo E., Josan A., Liu M., Neuhoff D.L., Pradhan S.S. The effect of node density and propagation model on throughput scaling of wireless networks. Proceedings of the 2006 IEEE International Symposium on Information Theory.

[14] Franceschetti M., Dousse O., Tse D.N.C., Thiran P. (2007). Closing the gap in the capacity of wireless networks via percolation theory. IEEE Trans. Inf. Theory.

[15] Grossglauser M., Tse D.N.C. (2002). Mobility increases the capacity of ad hoc wireless networks. IEEE ACM Trans. Netw..

[16] Neely M.J., Modiano E. (2005). Capacity and delay tradeoffs for ad hoc mobile networks. IEEE Trans. Inf. Theory.

[17] Gamal A.E., Mammen J., Prabhakar B., Shah D. Throughput-delay trade-off in wireless networks. Proceedings of the Twenty-Third Annual Joint Conference of the IEEE Computer and Communications Societies.

[18] Gamal A.E., Mammen J., Prabhakar B., Shah D. (2006). Optimal throughput-delay scaling in wireless networks—Part I: The fluid model. IEEE Trans. Inf. Theory.

[19] Gamal A.E., Mammen J., Prabhakar B., Shah D. Throughput-delay scaling in wireless networks with constant-size packets. Proceedings of the 2005 International Symposium on Information Theory.

[20] Lin X., Sharma G., Mazumdar R.R., Shroff N.B. (2006). Degenerate delay-capacity tradeoffs in ad-hoc networks with Brownian mobility. IEEE Trans. Inf. Theory.

[21] Kim Y., Lee K., Shroff N.B., Rhee I. Revisiting delay-capacity tradeoffs for mobile networks: The delay is overestimated. Proceedings of the 2012 IEEE Conference on Computer Communications.

[22] Lee K., Kim Y., Chong S., Rhee I., Yi Y., Shroff N.B. (2013). On the critical delays of mobile networks under levy walks and levy flights. IEEE ACM Trans. Netw..

[23] Liu B., Liu Z., Towsley D. On the capacity of hybrid wireless networks. Proceedings of the Twenty-Second Annual Joint Conference of the IEEE Computer and Communications.

[24] Liu B., Thiran P., Towsley D. Capacity of a wireless ad hoc network with infrastructure. Proceedings of the 8th ACM International Symposium on Mobile Ad Hoc Networking and Computing.

[25] Zemlianov A., de Veciana G. (2005). Capacity of ad hoc wireless networks with infrastructure support. IEEE J. Sel. Areas Commun..

[26] Toumpis S. Capacity bounds for three classes of wireless networks: Asymmetric, cluster, and hybrid. Proceedings of the 5th ACM International Symposium on Mobile Ad Hoc Networking and Computing.

[27] Kozat U.C., Tassiulas L. Throughput capacity of random ad hoc networks with infrastructure Support. Proceedings of the 9th Annual International Conference on Mobile Computing and Networking.

[28] Agarwal A., Kumar P.R. (2004). Capacity bounds for ad hoc and hybrid wireless networks. SIGCOMM Comput. Commun. Rev..

[29] Li P., Zhang C., Fang Y. (2009). Capacity and delay of hybrid wireless broadband access networks. IEEE J. Sel. Areas Commun..

[30] Huang W., Wang X., Zhang Q. Capacity scaling in mobile wireless ad hoc network with infrastructure support. Proceedings of the IEEE 30th International Conference on Distributed Computing Systems.

[31] Fu L., Yang S., Wang X., Gan X. Capacity and delay tradeoffs of motionCast with base stations. Proceedings of the 2011 IEEE Global Telecommunications Conference.

[32] Wang X., Huang W., Wang S., Zhang J., Hu C. (2011). Delay and capacity tradeoff analysis for motion cast. IEEE ACM Trans. Netw..

[33] Wang Y., Chu X., Wang X., Cheng Y. Optimal multicast capacity and delay tradeoffs in MANETs: A global perspective. Proceedings of the 2011 IEEE International Conference on Computer Communications.

[34] Zhang J., Wang X., Tian X., Wang Y., Chu X., Cheng Y. (2014). Optimal multicast capacity and delay tradeoffs in MANETs. IEEE Trans. Mob. Comput..

[35] Zhang J., Li Y., Liu Z., Wu F., Yang F., Wang X. (2015). On multicast capacity and delay in cognitive radio mobile ad hoc networks. IEEE Trans. Wirel. Commun..

[36] Luo J., Zhang J., Yu L., Wang X. (2015). The role of location popularity in multicast mobile ad hoc networks. IEEE Trans. Wirel. Commun..

[37] Wang X., Fu L., Tian X., Bei Y., Peng Q., Gan X., Yu H., Liu J. (2012). Converge cast: On the capacity and delay tradeoffs. IEEE Trans. Mob. Comput..

[38] Liu S., Yang F., Gan X., Tian X., Wang X., Liu J. Capacity and delay tradeoff in correlated hybrid Ad-Hoc networks. Proceedings of the 2014 IEEE Global Communications Conference.

[39] Wang C., Ye B., Wang X., Guo S., Lu S. (2014). Delay and capacity analysis in MANETs with correlated mobility and *f*-cast relay. IEEE Trans. Parallel Distrib. Syst..

[40] Wang C., Li X.Y., Jiang C., Yan H. (2014). The impact of rate adaptation on capacity-delay tradeoffs in mobile ad doc networks. IEEE Trans. Mob. Comput..

[41] Liu J., Nishiyama H., Kato N., Ma J.F., Jiang X. Throughput-delay tradeoff in mobile ad hoc networks with correlated mobility. Proceedings of the 2014 IEEE Conference on Computer Communications.

[42] Luo J., Zhang J., Yu L., Wang X. (2015). Impact of location popularity on throughput and delay in mobile ad hoc networks. IEEE Trans. Mob. Comput..

[43] Liu J., Kato N., Ma J., Sakano T. (2015). Throughput and delay tradeoffs for mobile ad hoc networks with reference point group mobility. IEEE Trans. Wirel. Commun..

[44] Qin Y., Li Y., Wu W., Yang F., Wang X., Xu J. (2015). Near-optimal scheme for cognitive radio networks with heterogeneous mobile secondary users. IEEE Trans. Commun..

[45] Ma X., Li F., Liu J., Liu X. (2015). Throughput-delay tradeoff for wireless multichannel multi-interface random networks. Can. J. Electr. Comput. Eng..

[46] Zhang X.M., Zhang Y., Yan F., Vasilakos A.V. (2015). Interference-Based Topology Control Algorithm for Delay-Constrained Mobile Ad Hoc Networks. IEEE Trans. Mob. Comput..

[47] Hu Y., Liu D., Wu Y. A new distributed topology control algorithm based on optimization of delay in ad hoc networks. Proceedings of the 2016 First IEEE International Conference on Computer Communication and the Internet.

[48] Ye H., Liu C., Hong X., Shi H. Uplink capacity-delay trade-off in hybrid cellular D2D networks with user collaboration. Proceeding of the International Symposium on Wireless Personal Multimedia Communications.

[49] Mattfeldt T. (1996). Stochastic Geometry and Its Applications.

[50] Andrews J.G., Baccelli F., Ganti R.K. (2011). A tractable approach to coverage and rate in cellular networks. IEEE Trans. Commun..

[51] Yu S.M., Kim S.L. Downlink capacity and base station density in cellular networks. Proceedings of the 11th International Symposium on Modeling Optimization in Mobile, Ad Hoc Wireless Networks.

[52] Ganesh A., O’Connell N., Wischik D. (1982). The Single Server Queue.

[53] Dong M., Ota K., Liu A. (2016). RMER: Reliable and Energy-Efficient Data Collection for Large-Scale Wireless Sensor Networks. IEEE Internet Things J..

[54] Liu Y., Dong M., Ota K., Liu A. (2016). ActiveTrust: Secure and Trustable Routing in Wireless Sensor Networks. IEEE Trans. Inf. Forensics Secur..

[55] Hu Y., Dong M., Ota K., Liu A., Guo M. (2016). Mobile Target Detection in Wireless Sensor Networks with Adjustable Sensing Frequency. IEEE Syst. J..

